# Supplementary material for: Structure and regulation of full-length human leucine-rich repeat kinase 1
Source: Nat Commun. 2023 Aug 9;14:4797. doi: 10.1038/s41467-023-40532-2 (PMC10412621; doi:10.1038/s41467-023-40532-2)
Supplement: Supplementary file 1 — Supplementary Information [file 41467_2023_40532_MOESM1_ESM.pdf]

Supplementary Material for:

Structure and regulation of full-length human leucine-rich repeat kinase 1

Riley D. Metcalfe [1], Juliana A. Martinez Fiesco [1], Luis Bonet-Ponce [2], Jillian H. Kluss [2], Mark R. Cookson [2], Ping Zhang [1]*

[1]Center for Structural Biology, Center for Cancer Research, National Cancer Institute, Frederick, MD, 21702, USA
[2]Cell Biology and Gene Expression Section, National Institute on Aging, National Institutes of Health, Bethesda, MD 20892

*For correspondence, ping.zhang@nih.gov

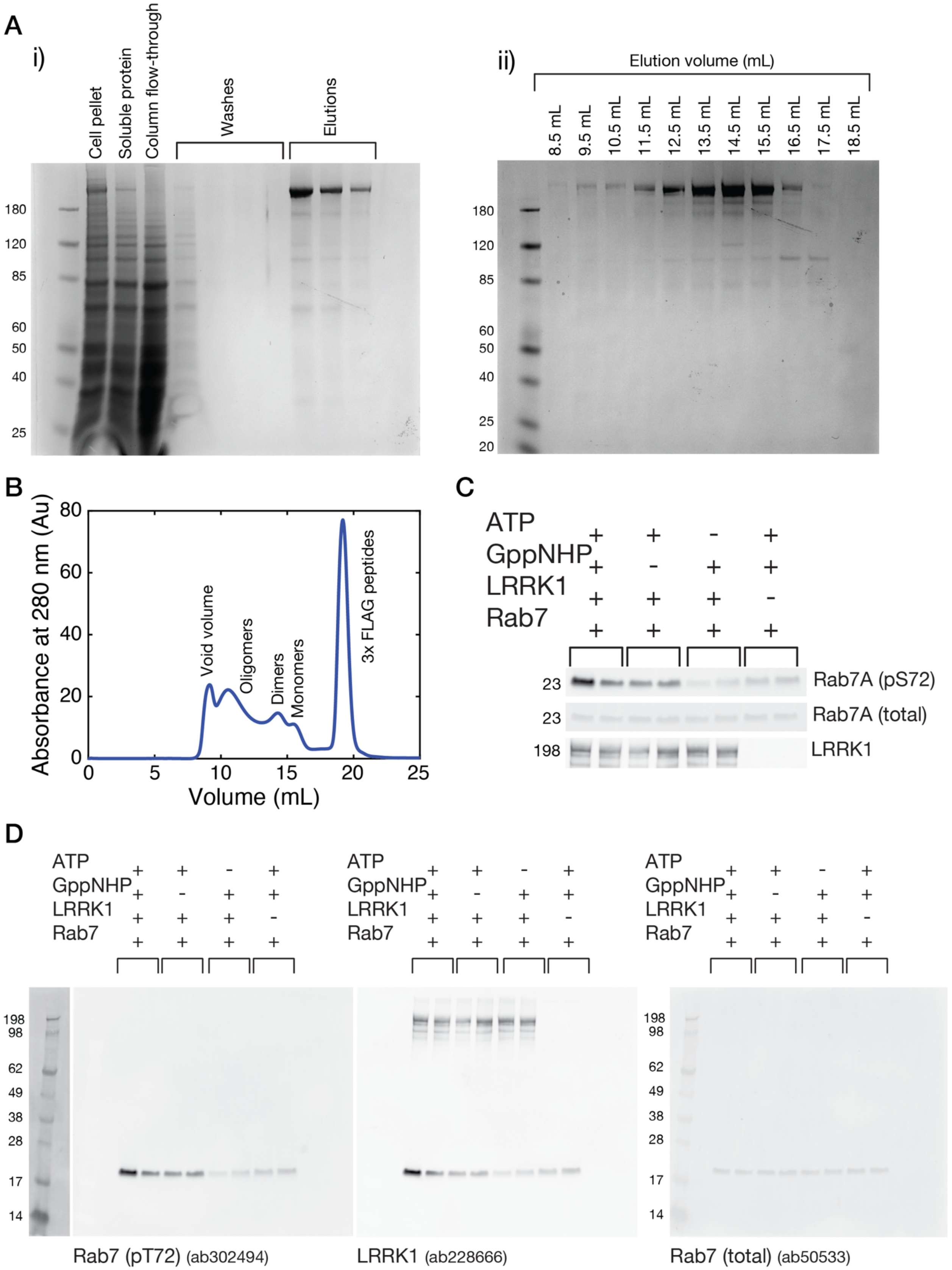
A
i)
Cell pellet
Soluble protein
Column flow-through
Washes
Elutions
180
120
85
60
50
40
25
ii)
Elution volume (mL)
8.5 mL
9.5 mL
10.5 mL
11.5 mL
12.5 mL
13.5 mL
14.5 mL
15.5 mL
16.5 mL
17.5 mL
18.5 mL
180
120
85
60
50
40
25
20
B
Absorbance at 280 nm (Au)
Volume (mL)
Void volume
Oligomers
Dimers
Monomers
3x FLAG peptides
C
ATP + + - +
GppNHP + - + +
LRRK1 + + + -
Rab7 + + + +
23
Rab7A (pS72)
23
Rab7A (total)
198
LRRK1
D
ATP + + - +
GppNHP + - + +
LRRK1 + + + -
Rab7 + + + +
198
98
62
49
38
28
17
14
Rab7 (pT72) (ab302494)
LRRK1 (ab228666)
Rab7 (total) (ab50533)

Supplementary Figure 1: Purification of LRRK1. A) 4-12% SDS-PAGE gels showing LRRK1 purity following, i) Flag-affinity purification and ii) gel filtration chromatography on a Superose 6 Increase 10/30 gel filtration chromatography column. B) Gel filtration chromatogram following Flag-affinity chromatography. Peaks corresponding to the void volume, high-molecular weight oligomers, dimers and monomers are indicated. Results are representative of multiple purifications conducted over the course of the structure-solution process. C) Western blot showing phosphorylation of the LRRK1 substrate Rab7A by the purified LRRK1. Results are representative of multiple assays conducted to confirm the LRRK1 kinase activity of the purified protein over the course of the structure-solution process. D) Complete membrane images for the Western blots shown in Supplementary Figure 1C.

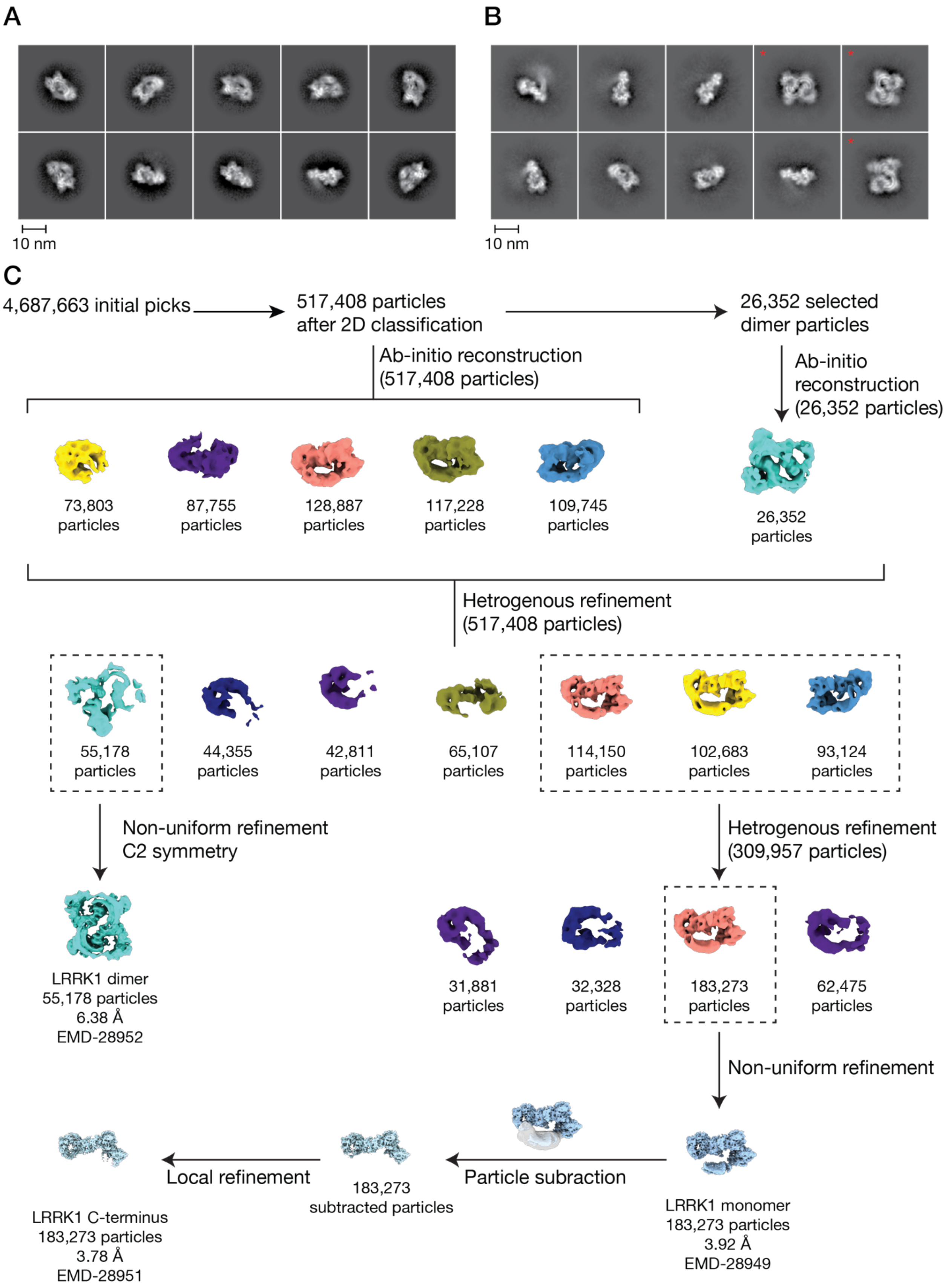
A
10 nm
B
10 nm
C
4,687,663 initial picks
517,408 particles after 2D classification
26,352 selected dimer particles
Ab-initio reconstruction (517,408 particles)
Ab-initio reconstruction (26,352 particles)
73,803 particles
87,755 particles
128,887 particles
117,228 particles
109,745 particles
26,352 particles
Hetrogenous refinement (517,408 particles)
55,178 particles
44,355 particles
42,811 particles
65,107 particles
114,150 particles
102,683 particles
93,124 particles
Non-uniform refinement C2 symmetry
Hetrogenous refinement (309,957 particles)
LRRK1 dimer
55,178 particles
6.38 Å
EMD-28952
31,881 particles
32,328 particles
183,273 particles
62,475 particles
Non-uniform refinement
Local refinement
Particle subraction
183,273 subtracted particles
LRRK1 C-terminus
183,273 particles
3.78 Å
EMD-28951
LRRK1 monomer
183,273 particles
3.92 Å
EMD-28949

Supplementary Figure 2: Cryo-EM data collection and processing for LRRK1. A) Representative 2D class averages for data collected from LRRK1 monomer grids. B) Representative 2D class averages for data collected from LRRK1 dimer grids. Representative 2D classes used for the reconstruction of the initial LRRK1 dimer volume are indicated with an asterisk. C) Summary of cryo-EM 3D reconstruction and refinement.

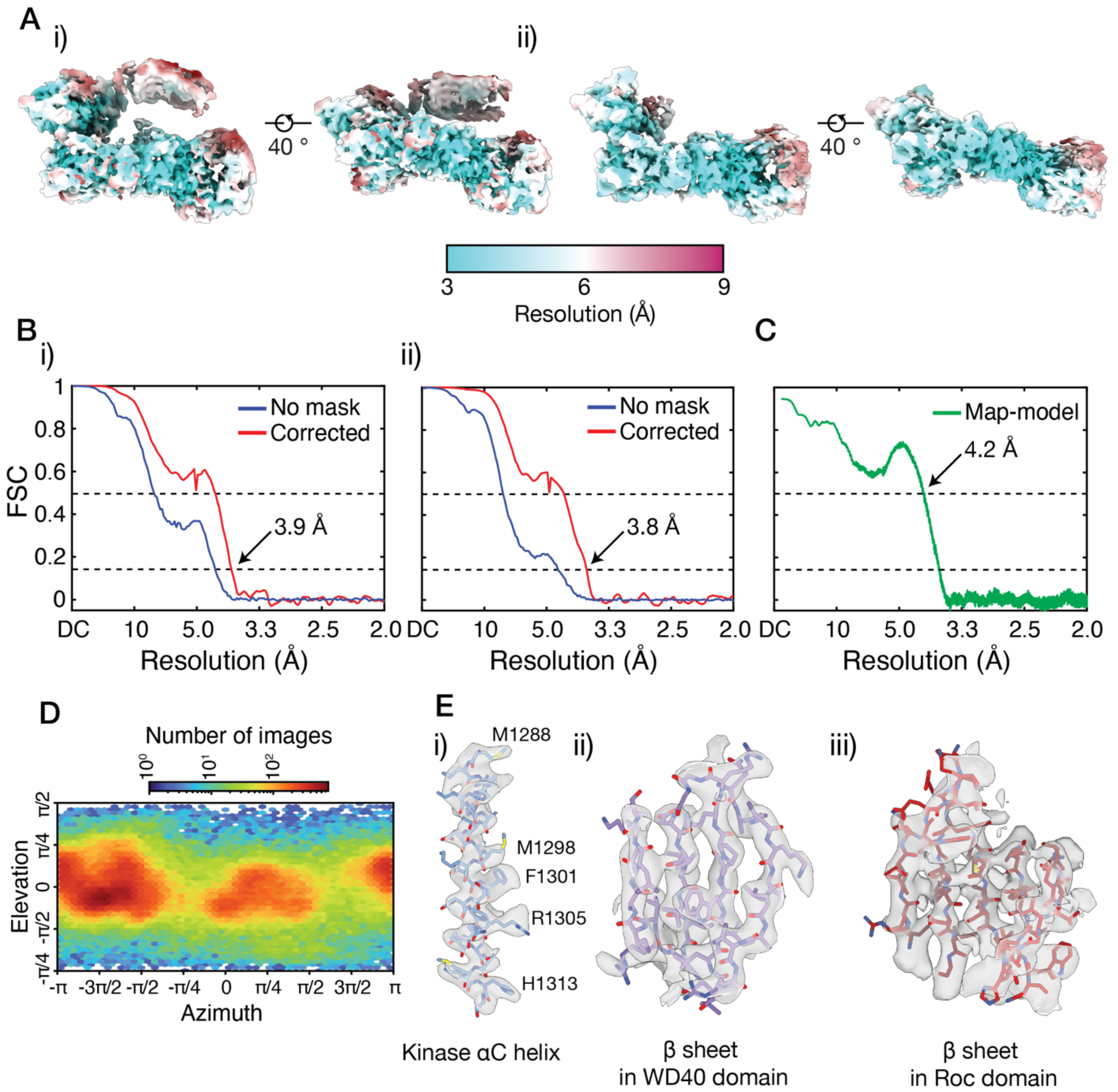

Supplementary Figure 3: Cryo-EM resolution estimation for the LRRK1 monomer. A) Local resolution maps for i) the global non-uniform refinement map, ii) the local refinement map for the RCKW domains. B) Gold-standard Fourier shell correlation (FSC) curves for, i) the global non-uniform refinement map, ii) the local refinement map. C) Map-model FSC curve. D) Viewing direction distribution for the global non-uniform refinement map. E) Representative density for the, i) kinase αC helix, ii) a β-sheet in the WD40 domain, iii) a β sheet in the Roc domain.

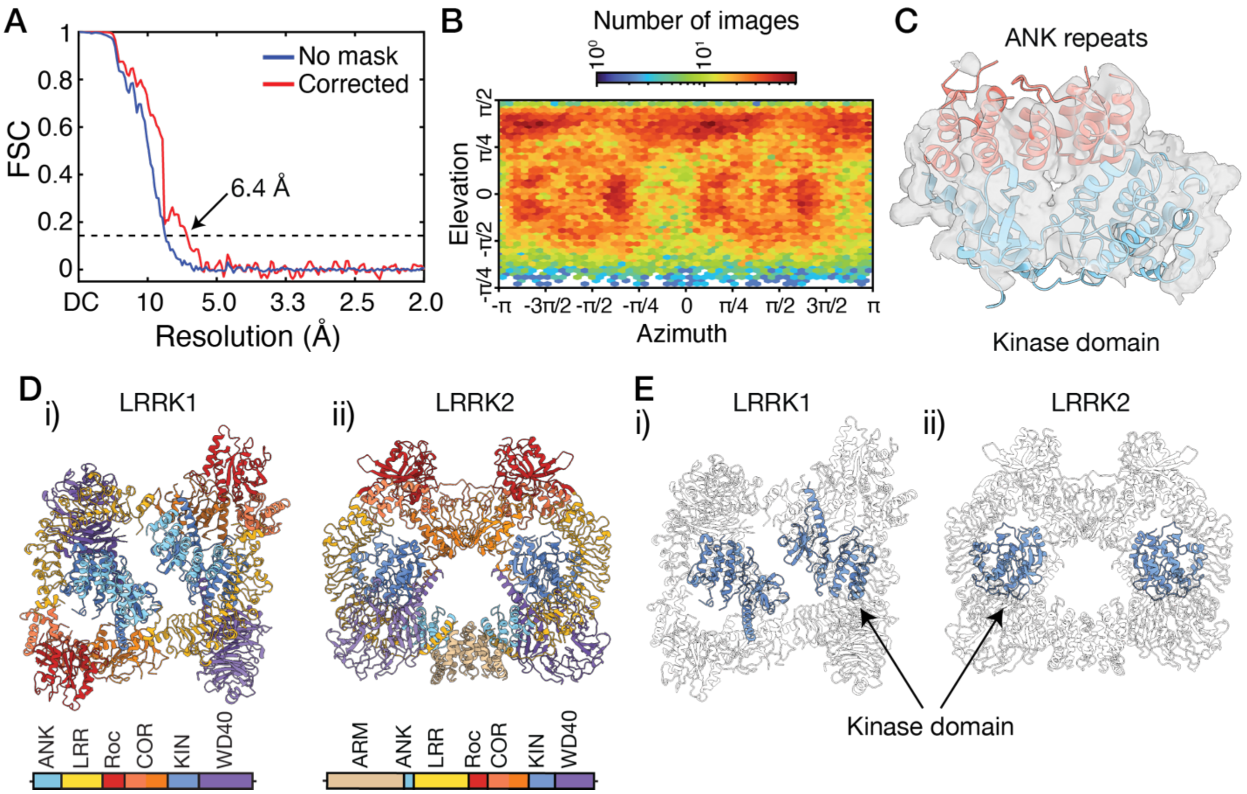

Supplementary Figure 4: Cryo-EM analysis of the LRRK1 dimer. A) Gold-standard FSC curve. B) Viewing direction distribution for the global non-uniform refinement map. C) Additional density observed above the kinase domain, assigned to the ankyrin repeats from the opposing molecule in the dimer. D) Comparison of the i), LRRK1 and ii), LRRK2 dimers. E) Comparison of the position of the kinase domain in the i), LRRK1 and ii), LRRK2 dimers.

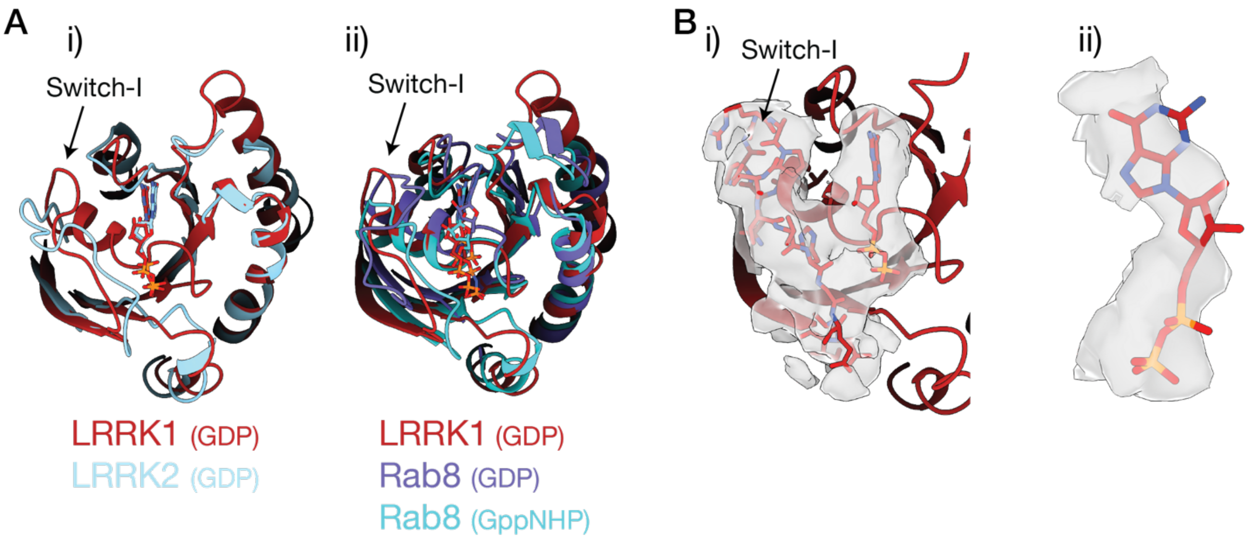

Supplementary Figure 5: The nucleotide state of the Roc domain. A) shows an overlay of the LRRK1 Roc domain with the Roc domain from i) LRRK2, bound to GDP (PDB: 7HLW[1]), ii) the small GTPase Rab8 bound to GDP (PDB: 4LHV[2]) and Rab8 bound to GppNHP (PDB: 4LHW), showing that the Switch-I motif in LRRK1 adopts a position consistent with GDP binding. B) i) Density supporting the position of GDP, the Switch-I motif and nearby residues, ii) Density supporting GDP.

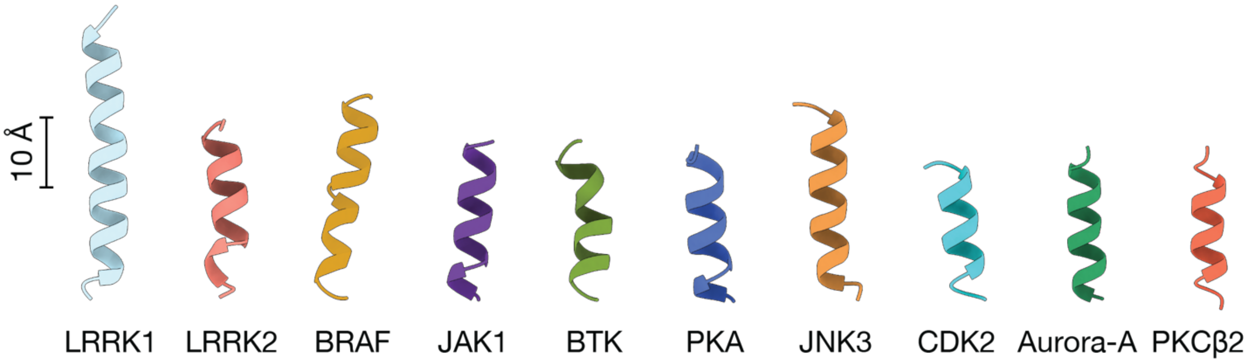

Supplementary Figure 6: Comparison of the αC helix from LRRK1 with other protein kinases. Alongside LRRK1, the αC helix from LRRK2 (PDB: 7LHW[1]), BRAF (PDB: 7MFD[3]), JAK1 (PDB: 6C7Y[4]), BTK (PDB: 1K2P[5]), PKA (PDB: 2CPK[6]), JNK3 (PDB: 1JNK[7]), CDK (PDB: 1FIN[8]), Aurora-A kinase (PDB: 1MQ4[9]) and PKCβ2 (PDB: 3PFQ[10]). Each αC helix is shown on the same scale, indicating that the LRRK1 helix is atypically long.

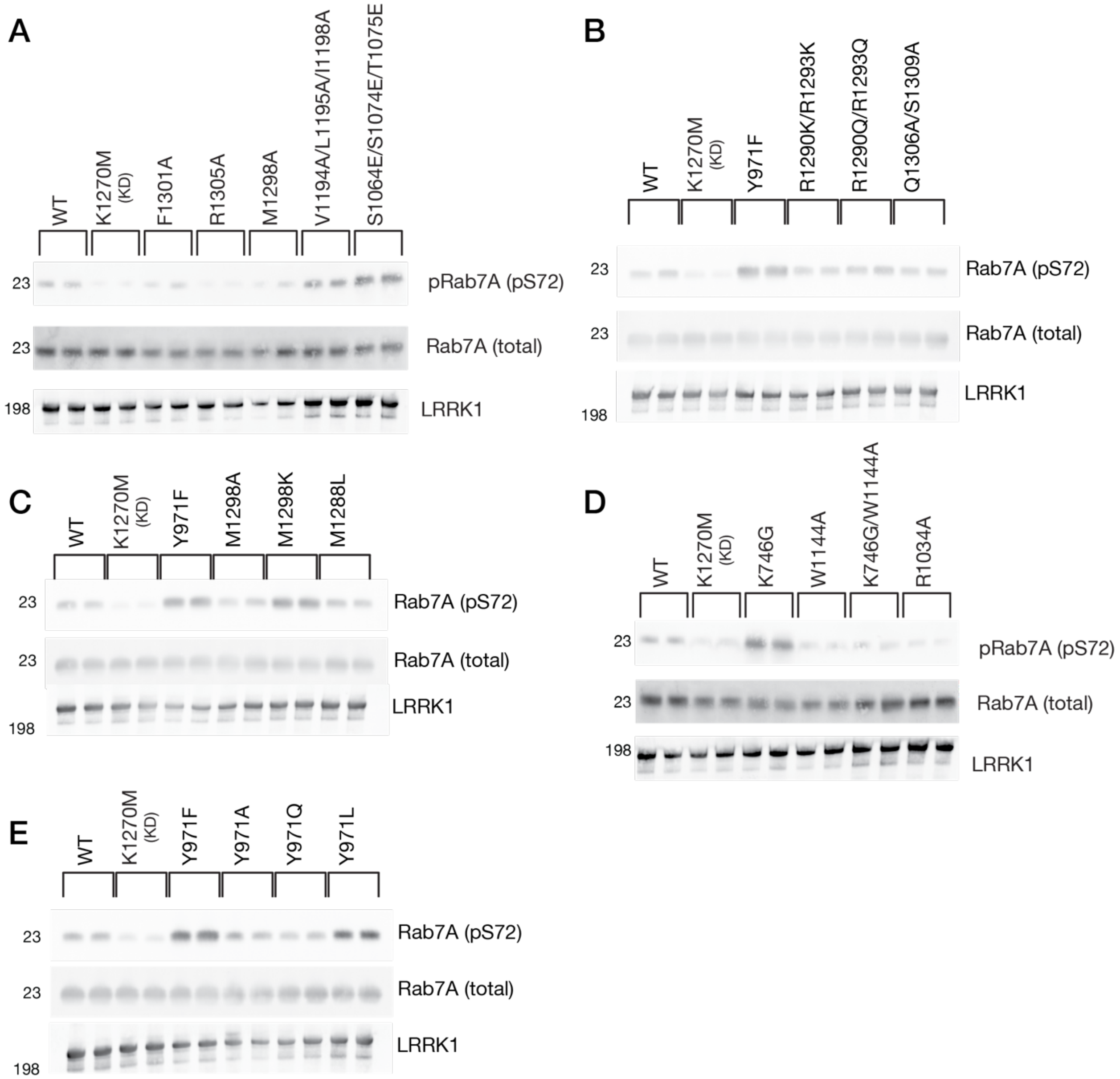

Supplementary Figure 7: Representative cropped membrane images for LRRK1 kinase assays. A-C) Related to Figure 4A, showing representative data for the results presented in this figure panel. D) Related to Figure 5A, showing representative data for the results presented in this figure panel. E) Related to Figure 6A, showing representative data for the results presented in this figure panel. For uncropped blot images, see Supplementary Figure 8 and Supplementary Figure 9.

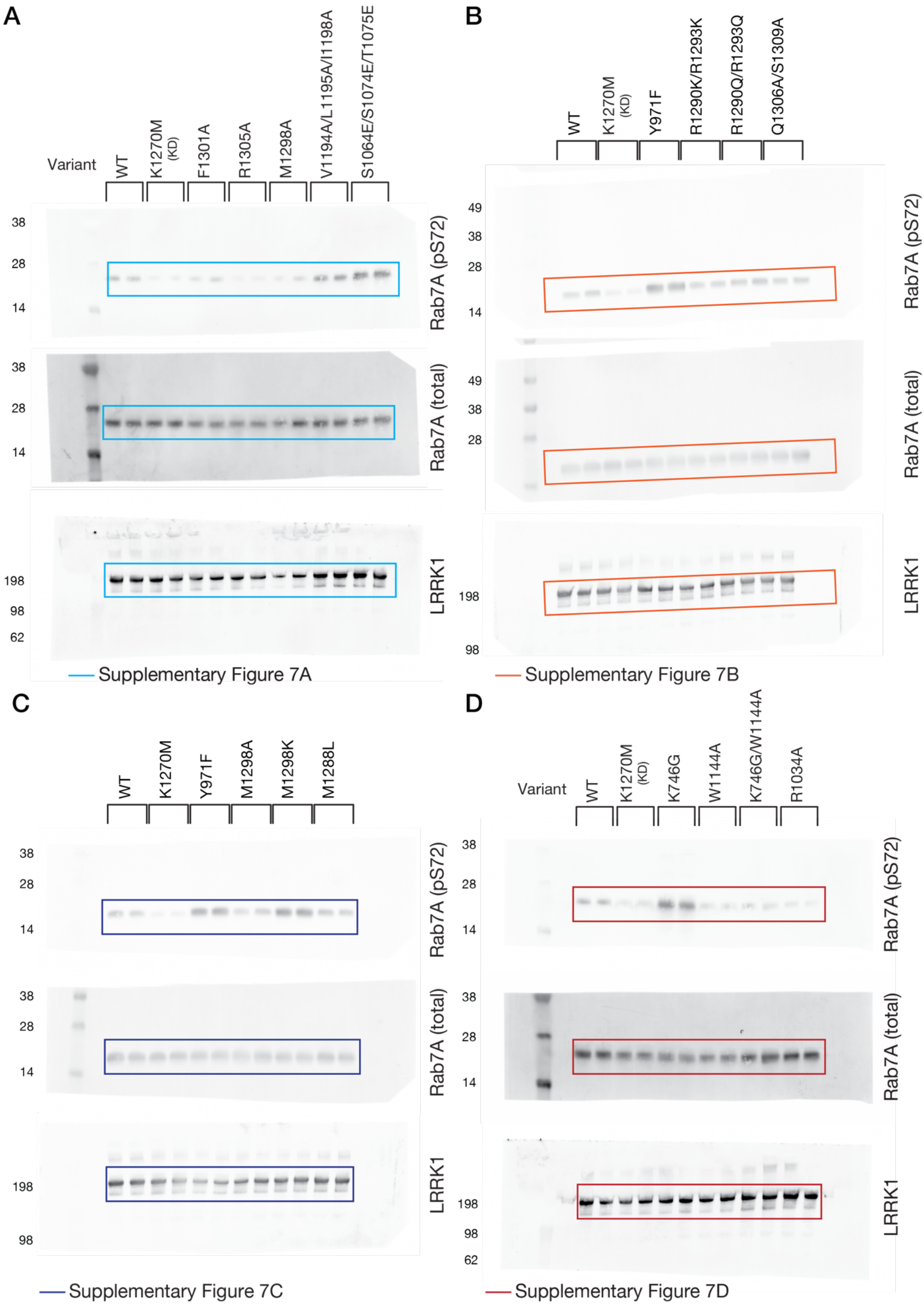
A
Variant
WT
K1270M (KD)
F1301A
R1305A
M1298A
V1194A/L1195A/I1198A
S1064E/S1074E/T1075E
38
28
14
Rab7A (pS72)
Rab7A (total)
LRRK1
198
98
62
Supplementary Figure 7A
B
WT
K1270M (KD)
Y971F
R1290K/R1293K
R1290Q/R1293Q
Q1306A/S1309A
49
Supplementary Figure 7B
C
K1270M
M1298K
M1288L
Supplementary Figure 7C
D
K746G
W1144A
K746G/W1144A
R1034A
Supplementary Figure 7D

Supplementary Figure 8: Uncropped Western blot membrane images for, A) the results presented in Supplementary Figure 7A, B) the results presented in Supplementary Figure 7B, C) the results presented in Supplementary Figure 7C, D) the results presented in Supplementary Figure 7D.

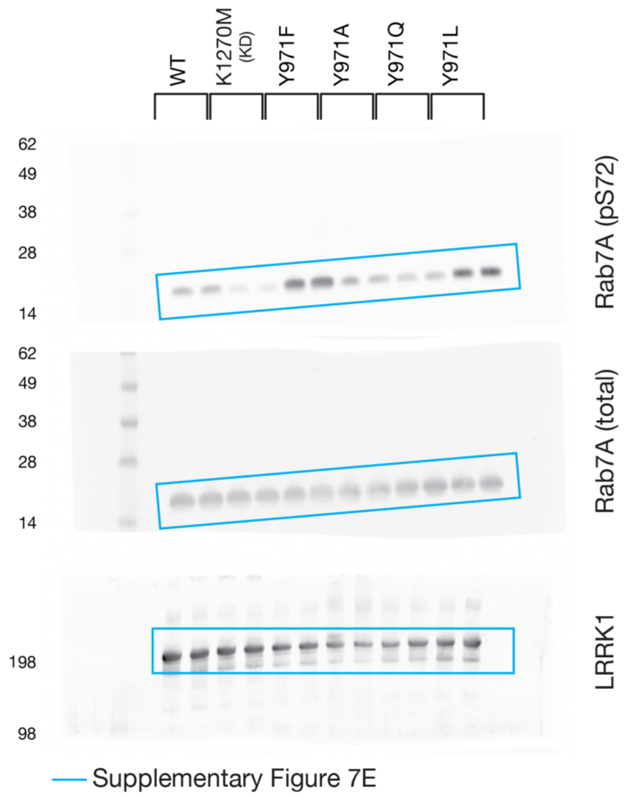

Supplementary Figure 9: Uncropped Western blot membrane images for the results presented in Supplementary Figure 7E.

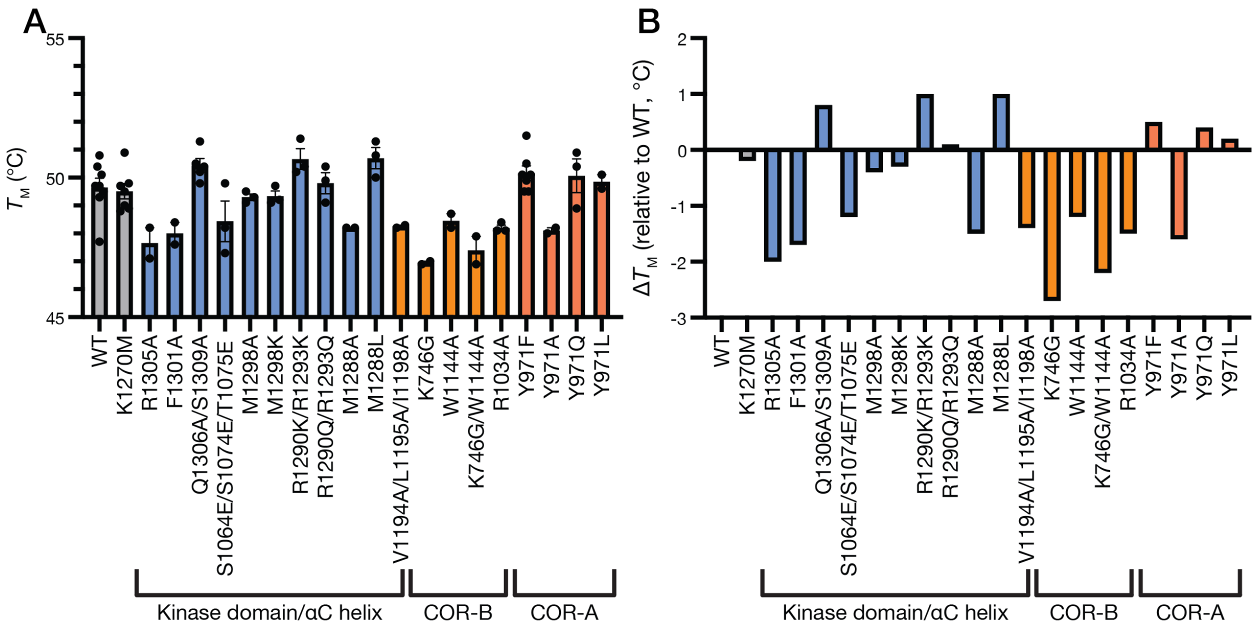

Supplementary Figure 10: Thermal stability of LRRK1 variants. A) Melting temperature (T_M) for the LRRK1 variants, measured using a Nanotemper Nano-DSF instrument. Error bars shown are mean ± SEM, n = 2-8 independent experiments. B) Melting temperature relative to WT LRRK1. Source data is provided in the source data file.

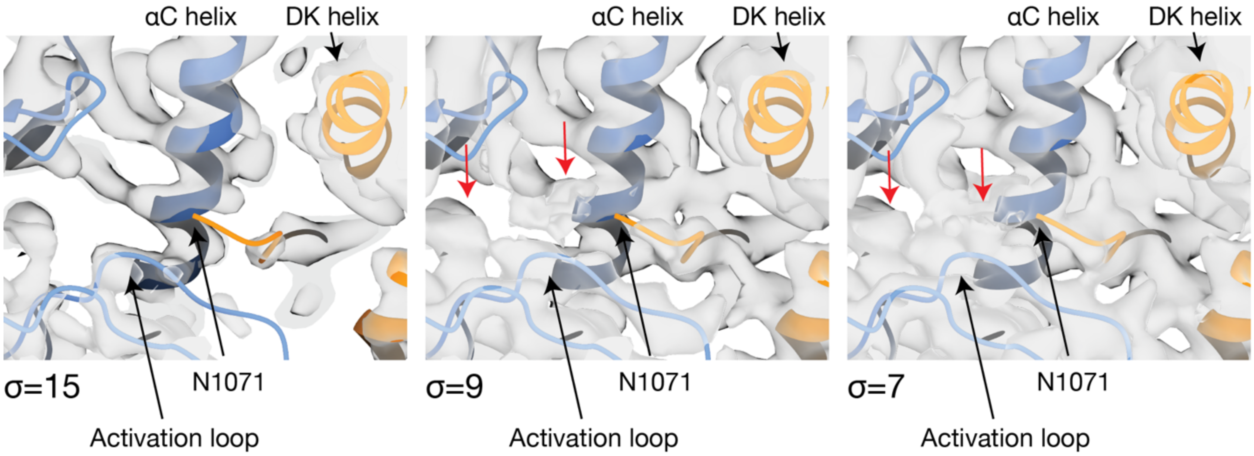

Supplementary Figure 11: Density for the loop in the COR-B domain which is phosphorylated by PKC. Contouring the density at a low level suggests that the loop continues past the αC helix and towards the kinase N-lobe.

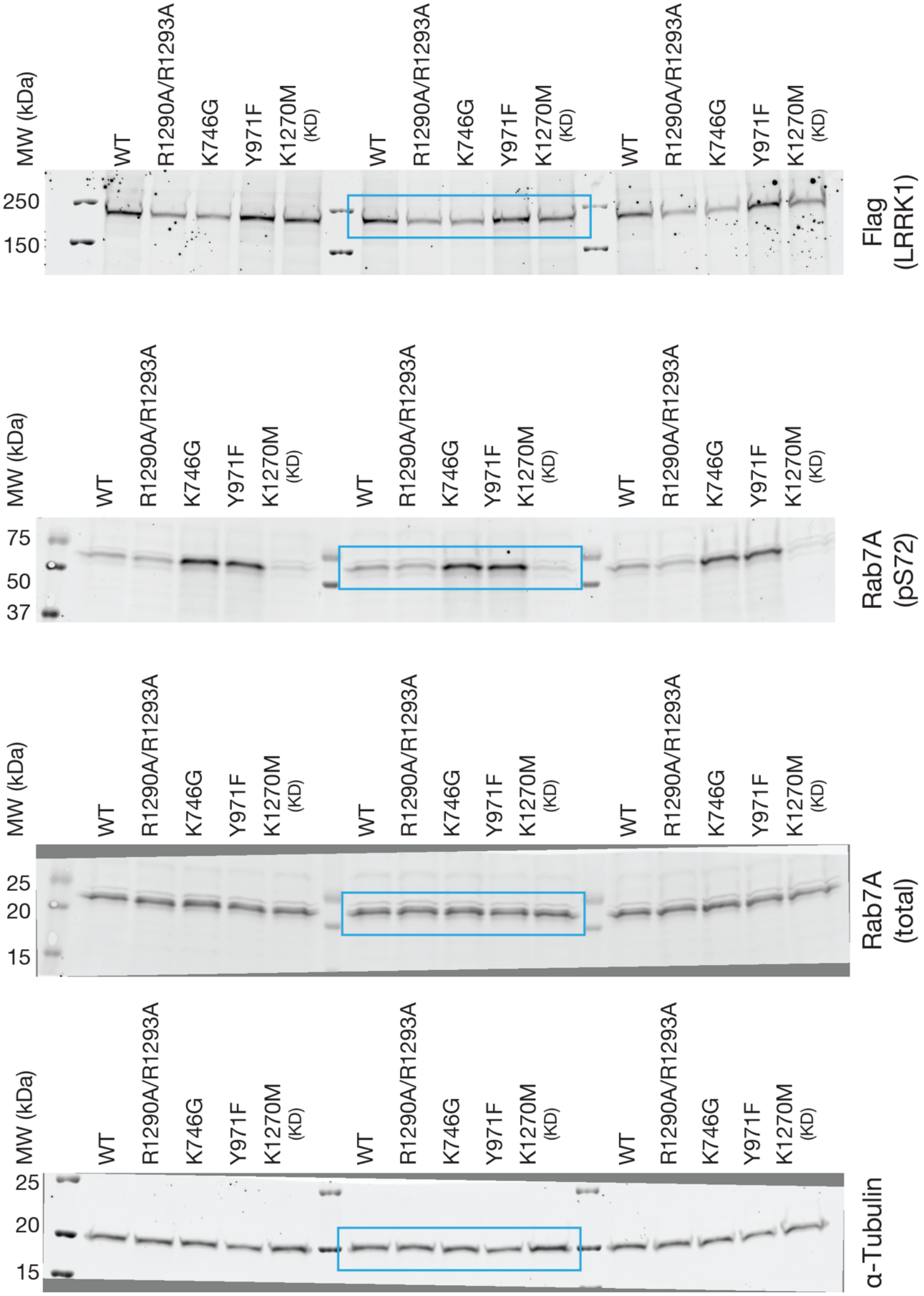

Supplementary Figure 12: Uncropped Western blot membrane images for the results presented in Figure 7A.

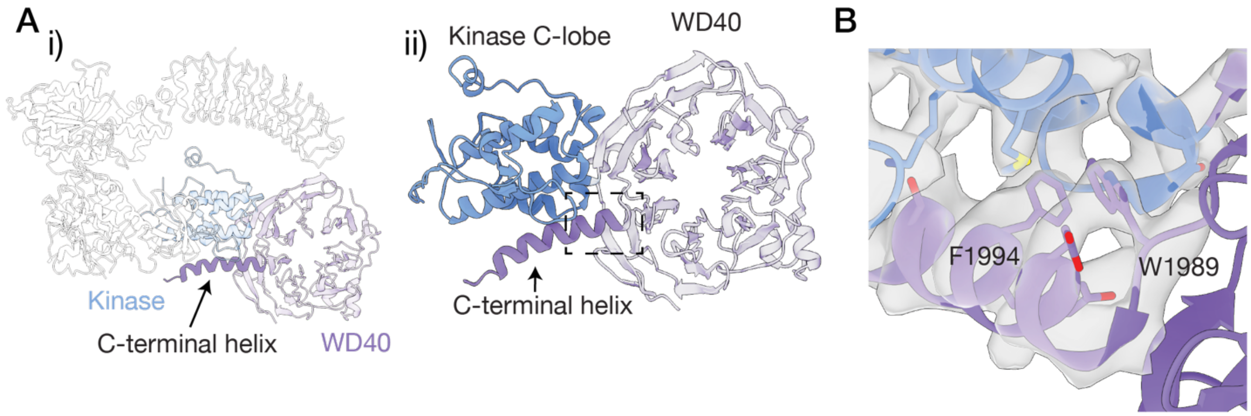

Supplementary Figure 13: The LRRK1 C-terminal helix. A) The structure of LRRK1 with the C-terminal helix indicated, i) the overall structure of LRRK1, ii) detail of the kinase C-lobe/WD40/C-terminal helix interface, highlighting the additional helix contributed to the kinase C-lobe fold contributed by the C-terminal helix. The area inside the dashed box is highlighted in B). B) Detail of the cryo-EM density, showing the position of W1989, the deletion of which causes OSMD.

Supplementary Table 1: Cryo-EM data collection, 3D reconstruction and model building statistics.

	LRRK1 monomer	LRRK1 C-terminus (LRRs subtracted, local refinement)	LRRK1 dimer
**Data collection and image processing**			
Microscope	Talos Arctica		
Camera	K3		
Magnification	100,000		
Electron energy (kV)	200		
Electron exposure ($e^{-}A^{-2}$)	50		
Defocus range (μm)	0.8-2.5		
Physical pixel size	0.81		
Total number of micrographs	32,157 (22,865 from monomer grids, 9,992 from dimer grids)		
Selected micrographs	26,686 (18,074 from monomer grids, 8,612 from dimer grids)		
Initial particle images	4,687,663		
Final particle images	183,273		55,178
Starting model	*De novo*		*De novo*
Map resolution	3.92	3.78	6.38
FSC threshold	0.132	0.132	0.132
Sharpening *B* factor ($Å^{-2}$)	129.2	104.3	402.9
EMDB code	EMD-28949	EMD-28951	EMD-28952
**Model building and refinement**			
Initial models used	AF-Q38SD2-F1		
Map used for refinement	Composite map, EMD-28950		
Model resolution	4.2		
FSC threshold	0.5		
Model composition			
Non-hydrogen atoms	10955		
Amino acid residues	1377		
Protein molecules	1		
Real-space correlation			
CCvolume	0.70		
CCmask	0.73		
Mean *B* factor ($Å^2$)	66.09		
RMS deviations			
Bond lengths (Å) (outliers >4σ)	0.002 (0)		
Bond angles (°) (outliers >4σ)	0.620 (1)		

Validation	
MOLProbity score	2.06
Clashscore	12.07
Rotamer outliers (%)	0.00
CaBLAM outliers (%)	4.60
Cβ outliers (%)	0.00
Ramachandran plot	
Favored (%)	92.54
Allowed (%)	7.46
Outliers (%)	0.00
PDB code	8FAC

Supplementary Material References:

1 Myasnikov, A. *et al.* Structural analysis of the full-length human LRRK2. *Cell*, 1-9, doi:10.1016/j.cell.2021.05.004 (2021).

2 Guo, Z., Hou, X., Goody, R. S. & Itzen, A. Intermediates in the guanine nucleotide exchange reaction of Rab8 protein catalyzed by guanine nucleotide exchange factors Rabin8 and GRAB. *Journal of Biological Chemistry* **288**, 32466-32474, doi:10.1074/jbc.M113.498329 (2013).

3 Martinez Fiesco, J. A., Durrant, D. E., Morrison, D. K. & Zhang, P. Structural insights into the BRAF monomer-to-dimer transition mediated by RAS binding. *Nature Communications* **13**, 1-14, doi:10.1038/s41467-022-28084-3 (2022).

4 Liau, N. P. D. *et al.* The molecular basis of JAK/STAT inhibition by SOCS1. *Nature Communications* **9**, 1-14, doi:10.1038/s41467-018-04013-1 (2018).

5 Mao, C., Zhou, M. & Uckun, F. M. Crystal Structure of Bruton's Tyrosine Kinase Domain Suggests a Novel Pathway for Activation and Provides Insights into the Molecular Basis of X-linked Agammaglobulinemia. *Journal of Biological Chemistry* **276**, 41435-41443, doi:10.1074/jbc.M104828200 (2001).

6 Knighton, D. R. *et al.* Crystal structure of the catalytic subunit of cyclic adenosine monophosphate-dependent protein kinase. *Science* **253**, 407-414, doi:10.1126/science.1862342 (1991).

7 Xie, X. *et al.* Crystal structure of JNK3: A kinase implicated in neuronal apoptosis. *Structure* **6**, 983-991, doi:10.1016/S0969-2126(98)00100-2 (1998).

8 Jeffrey, P. D. *et al.* Mechanism of CDK activation revealed by the structure of a cyclinA-CDK2 complex. *Nature* **376**, 313-320, doi:10.1038/376313a0 (1995).

9 Nowakowski, J. *et al.* Structures of the cancer-related Aurora-A, FAK, and EphA2 protein kinases from nanovolume crystallography. *Structure* **10**, 1659-1667, doi:10.1016/S0969-2126(02)00907-3 (2002).

10 Leonard, T. A., Róycki, B., Saidi, L. F., Hummer, G. & Hurley, J. H. Crystal structure and allosteric activation of protein kinase C βII. *Cell* **144**, 55-66, doi:10.1016/j.cell.2010.12.013 (2011).

11 Reimer, J. M. *et al.* Structure of LRRK1 and mechanisms of autoinhibition and activation. *bioRxiv* **2022**, 2022.2011.2022.517582 (2022).
