# Supplementary material for: Structure and regulation of full-length human leucine-rich repeat kinase 1
Source: Nat Commun. 2023 Aug 9;14:4797. doi: 10.1038/s41467-023-40532-2 (PMC10412621; doi:10.1038/s41467-023-40532-2)
Supplement: Supplementary file 3 — Description of additional supplementary files [file 41467_2023_40532_MOESM3_ESM.pdf]

# Description of additional supplementary files

**File Name: Supplementary Movie 1:**

Description: 3D variability analysis of LRRK1. Movie shows the three variability modes solved for LRRK1, using a filter resolution of 7 Å for solving and visualizing the components.
