# Supplementary material for: Structure and regulation of full-length human leucine-rich repeat kinase 1
Source: Nat Commun. 2023 Aug 9;14:4797. doi: 10.1038/s41467-023-40532-2 (PMC10412621; doi:10.1038/s41467-023-40532-2)
Supplement: Supplementary file 5 — Reporting Summary [file 41467_2023_40532_MOESM5_ESM.pdf]

nature portfolio

Corresponding author(s): Ping Zhang, NCI

Last updated by author(s): July 13, 2023

Reporting Summary

Nature Portfolio wishes to improve the reproducibility of the work that we publish. This form provides structure for consistency and transparency in reporting. For further information on Nature Portfolio policies, see our Editorial Policies and the Editorial Policy Checklist.

Statistics

For all statistical analyses, confirm that the following items are present in the figure legend, table legend, main text, or Methods section.

n/a	Confirmed	
☐	☒	The exact sample size (*n*) for each experimental group/condition, given as a discrete number and unit of measurement
☐	☒	A statement on whether measurements were taken from distinct samples or whether the same sample was measured repeatedly
☐	☒	The statistical test(s) used AND whether they are one- or two-sided<br>*Only common tests should be described solely by name; describe more complex techniques in the Methods section.*
☒	☐	A description of all covariates tested
☒	☐	A description of any assumptions or corrections, such as tests of normality and adjustment for multiple comparisons
☐	☒	A full description of the statistical parameters including central tendency (e.g. means) or other basic estimates (e.g. regression coefficient) AND variation (e.g. standard deviation) or associated estimates of uncertainty (e.g. confidence intervals)
☐	☒	For null hypothesis testing, the test statistic (e.g. *F*, *t*, *r*) with confidence intervals, effect sizes, degrees of freedom and *P* value noted<br>*Give P values as exact values whenever suitable.*
☒	☐	For Bayesian analysis, information on the choice of priors and Markov chain Monte Carlo settings
☒	☐	For hierarchical and complex designs, identification of the appropriate level for tests and full reporting of outcomes
☐	☒	Estimates of effect sizes (e.g. Cohen's *d*, Pearson's *r*), indicating how they were calculated

Our web collection on statistics for biologists contains articles on many of the points above.

Software and code

Policy information about availability of computer code

Data collection	EPU v2.14, AcquireMP 2022R1, ASTRA v8, PR ThermControl v.2.1.6, Typhoon control software v1.1.0.7.
Data analysis	Cryosparc v3.3, ASTRA v8, DiscoverMP 2022R1, Fiji 2.3, Phenix v.1.20, Microsoft Excel 16.75, Prism v.9.4.1, UCSF Chimera v1.15, UCSF ChimeraX v1.4, PR ThermControl v.2.1.6, deepEMhancer v. 0.13

For manuscripts utilizing custom algorithms or software that are central to the research but not yet described in published literature, software must be made available to editors and reviewers. We strongly encourage code deposition in a community repository (e.g. GitHub). See the Nature Portfolio guidelines for submitting code & software for further information.

Data

Policy information about availability of data

All manuscripts must include a data availability statement. This statement should provide the following information, where applicable:

- Accession codes, unique identifiers, or web links for publicly available datasets
- A description of any restrictions on data availability
- For clinical datasets or third party data, please ensure that the statement adheres to our policy

The cryo-EM maps generated in this study have been deposited in the EMDB database under accession code EMD-28950 (LRRK1 monomer composite map), EMD-28949, (LRRK1 monomer global refinement), EMD-28951 [https://www.ebi.ac.uk/pdbe/entry/emdb/EMD-28951] (LRRK1 C-terminus local refinement after subtraction of the LRRs), and EMD-28952 (LRRK1 dimer). The atomic model of the LRRK1 monomer has been deposited in the PDB with accession code 8FAC.The quantified kinase assay, confocal microscopy and differential scanning fluorimetry melting temperature data generated in this study are provided in the Source Data

file.

Human research participants

Policy information about studies involving human research participants and Sex and Gender in Research.

Reporting on sex and gender	N/A
Population characteristics	N/A
Recruitment	N/A
Ethics oversight	N/A

Note that full information on the approval of the study protocol must also be provided in the manuscript.

Field-specific reporting

Please select the one below that is the best fit for your research. If you are not sure, read the appropriate sections before making your selection.

☒ Life sciences ☐ Behavioural & social sciences ☐ Ecological, evolutionary & environmental sciences

For a reference copy of the document with all sections, see nature.com/documents/nr-reporting-summary-flat.pdf

Life sciences study design

All studies must disclose on these points even when the disclosure is negative.

Sample size	EM particle sample size was determined by automated particle picking and curation. The particles selected were sufficient to give a map with ~4 Å resolution, as determined by gold-standard FSC analysis, as is standard is the field.<br>Sample sizes for recombinant protein kinase assays, cellular assays and fluorescence microscopy assays were not predetermined. The sample sizes used were sufficient to represent the reproducibility of the assays and are in line with conventions in the field.
Data exclusions	Particles providing the highest quality 3D reconstruction were retained. Other particles were excluded. No data were excluded from biochemical experiments.
Replication	CryoEM density maps were calculated from thousands of particles and multiple (7) grids to obtain the final reconstructions. The structure determination process was not replicated. All kinase assays were replicated to confirm reproducibility using at least three independent protein preparations. In vitro kinase assays were replicated from three independent experiments, as indicated in the figure legend. Colocalization experiments were not replicated from multiple independent experiments.
Randomization	This is not relevant, as we do not have organisms or subjects that require (or allow) randomization.
Blinding	This is not relevant, as we do not have organisms or subjects that require blinding.

Reporting for specific materials, systems and methods

We require information from authors about some types of materials, experimental systems and methods used in many studies. Here, indicate whether each material, system or method listed is relevant to your study. If you are not sure if a list item applies to your research, read the appropriate section before selecting a response.

Materials & experimental systems

n/a	Involved in the study	
☐	☒	Antibodies
☐	☒	Eukaryotic cell lines
☒	☐	Palaeontology and archaeology
☒	☐	Animals and other organisms
☒	☐	Clinical data
☒	☐	Dual use research of concern

Methods

n/a	Involved in the study	
☒	☐	ChIP-seq
☒	☐	Flow cytometry
☒	☐	MRI-based neuroimaging

Antibodies

Antibodies used	Rab7 (total, mouse), Abcam cat. ab50533, Rab7 (pS72, rabbit), Abcam cat. ab302494, LRRK1 (total, rabbit), Abcam cat. ab228666, mouse anti-FLAG (Millipore-Sigma cat#F3165), mouse anti-Rab7 (Cell Signaling Technologies, cat#95746), mouse anti-atubulin (Cell Signaling Technologies, cat#3876), donkey anti-mouse secondary fluorescently labeled antibody Alexa Fluor™ 568, Thermo Fisher Scientific cat#A10037, goat anti-mouse IR-fluorescent secondary antibody (LiCor cat. 926-68072), goat anti-rabbit IR-fluorescent secondary antibody (LiCor cat. 926-3221).<br><br>Dilutions are noted in the manuscript.
Validation	A manuscript has been published describing in part the Rab7 (pS72) antibody (Malik et al. Biochem J 2021, PMID 33459343). The Abcam Rab7 (total) and Abcam LRRK1 (total) antibodies were validated by the manufacturer using cell lines that are known to express the protein, no knock-out validation was noted. The Cell Signaling Technologies Rab7 (total) and anti-atubulin, was validated by the manufacturer using cell lines that are known to express the protein, no knock-out validation was noted. No validation was noted for the Millipore-Sigma mouse anti-FLAG antibody.

Eukaryotic cell lines

Policy information about cell lines and Sex and Gender in Research

Cell line source(s)	Sf9 insect cells, Life Technologies cat. 11496015<br>U20S cells ATCC, cat #HTB-96
Authentication	Cell lines were not authenticated.
Mycoplasma contamination	Insect cells were not routinely tested for mycoplasma contamination. U20S cells were tested for mycoplasma contamination.
Commonly misidentified lines<br>(See ICLAC register)	No commonly misidentified lines were used.
